# AI in MRI: Computational Frameworks for a Faster, Optimized, and Automated Imaging Workflow

**DOI:** 10.3390/bioengineering10040492

**Published:** 2023-04-20

**Authors:** Efrat Shimron, Or Perlman

**Affiliations:** 1 Department of Electrical Engineering and Computer Sciences, University of California, Berkeley, CA 94720, USA; 2 Department of Biomedical Engineering, Tel Aviv University, Tel Aviv 6997801, Israel; 3 Sagol School of Neuroscience, Tel Aviv University, Tel Aviv 6997801, Israel

**Keywords:** artificial intelligence (AI), machine learning, magnetic resonance imaging (MRI), deep learning, image reconstruction, segmentation, medical imaging, acceleration

Over the last decade, artificial intelligence (AI) has made an enormous impact on a wide range of fields, including science, engineering, informatics, finance, and transportation. In recent years, it has also created new opportunities for improving the quality and efficiency of patient care. As the first breakthroughs in deep learning (DL) were largely concerned with image perception, interpretation, and analysis, it is not surprising that harnessing this technology has led to enormous progress in medical imaging. For example, DL techniques have enabled state-of-the-art results in image formation and analysis, pathology detection, and protocol planning [1,2,3,4,5,6,7,8,9,10,11].

This special issue of *Bioengineering*, entitled: “AI in MRI: Frontiers and Applications” focuses on the application of AI in magnetic resonance imaging (MRI), an area that has recently seen a surge of activity. MRI is an imaging modality that allows for the non-invasive visualization of the body’s internal structure and function, and is widely used for various clinical applications in areas including neurology, oncology, cardiology, and orthopedics, and in adult, pediatric, and fetal imaging. MRI has several advantages over other imaging modalities, including a lack of ionizing radiation, the ability to generate excellent soft-tissue contrast, and the ability to acquire images in any plane, orientation, and depth. Nevertheless, MRI is characterized by a few substantial limitations, primarily its relatively long scan time, which translates into high cost and increased sensitivity to motion artifacts. Recently, AI-based techniques have enabled considerable progress in addressing these limitations, providing accelerated acquisition [12,13,14,15,16] and motion robustness [17,18,19,20,21].

This special issue features seventeen papers that showcase the added value of employing AI-based solutions for a wide range of MRI-associated tasks, occurring at different points along the imaging pipeline. These original research reports can be divided into four main categories: MRI Acceleration, Image Synthesis and Parameter Quantification, Automated Segmentation, and Scan Planning.

## 1. MRI Acceleration

In the last few decades, extensive research efforts were dedicated to accelerating MRI via the development of advanced data sampling and reconstruction techniques [22,23,24,25,26,27,28,29,30,31,32,33]. Such techniques commonly involve rapid acquisition schemes that “break” the classical Nyquist-sampling criterion, a process known as *undersampling*. As this approach leads to image-domain artifacts, carefully designed reconstruction techniques are essential to allow for clinical-quality-preserving image recovery. Recently, DL techniques have enabled state-of-the-art results in this task, enabling high acceleration and excellent reconstruction quality [7,8,9,12,13,14,15,16,34,35,36,37,38,39]. Their success can be attributed to the ability to learn image priors in a data-driven manner instead of the hand-crafted manner practiced in compressed sensing and dictionary learning [25,29,35]. Furthermore, physics-guided unrolled neural networks combine the benefits of DL-based artifact-removal modules with data consistency blocks, which incorporate a physics-based model of the imaging system [9]. A large body of work has demonstrated the benefits of DL for image reconstruction in 2D MRI scans [7,8,9,34,35,36,37,38,39]. More recently, attention has shifted to harnessing DL for accelerating higher-dimensional MRI scans, such as dynamic (temporal) MRI. In this issue, Oscanoa et al. provide a comprehensive review of DL-based reconstruction methods for dynamic cardiac MRI, with connections to relevant theory [40].

One research direction that has seen a recent flair of activity is the development of AI techniques for the joint optimization of a non-Cartesian k-space sampling trajectory and an image-reconstruction network [41,42,43,44]. In this issue, Radhakrishna and Ciuciu [45] introduce a generic framework, dubbed projection for jointly learning non-Cartesian trajectories while optimizing reconstructor trajectories (PROJECTOR). This framework ensures that the learned trajectories are compatible with gradient-related hardware constraints. In contrast to previous techniques that enforce such constraints via penalty terms, PROJECTOR enforces them through embedded projection steps that project the learned trajectory on a feasible set. Retrospective experiments with 2D and 3D MRI data indicate that the PROJECTOR-generated trajectories can exploit the full possible range of gradients and slew rates well and produce sharp images. In another work, Hossain et al. [46] propose a new sampling pattern for 2D MRI, which combines the random and non-random sampling of the phase-encoding direction. The authors also introduce an advanced fully dense attention convolutional neural network (FDA-CNN), which reduces the number of redundant features using attention gates. The article by Cho et al. [47] proposes a different strategy for synergistic acquisition/reconstruction design. The authors propose to combine a wave-encoded sampling strategy with an unrolled neural network. Their strategy exploits the inherent similarity of images acquired with different contrasts of echo times, which can be used for accelerating quantitative MRI.

Here, several papers introduce techniques for developing DL models while facing data-related challenges. Zou et al. [48] introduce a new framework for dynamic MRI reconstruction without ground truth data, namely self-supervised collaborative learning (SelcCoLearn). This framework splits the undersampled k-space measurements into two datasets and uses them as inputs for two neural networks. Those networks have the same structure but different weights, and they are trained in parallel. The authors introduce a co-training loss that promotes the consistency of the predictions of the two networks. Experiments with cardiac data indicate that SelCoLearn produces high-quality reconstructions of dynamic MRI data. Additionally, Deveshwar et al. [49] introduce a method for synthesizing multi-coil complex-valued data from magnitude-only data; this can be useful for leveraging the high number of DICOM images that are stored in clinical databases. Their method uses conditional generative adversarial networks (GANs) for generating synthetic-phase images and ESPIRiT [28] for generating sensitivity maps from publicly available databases. The authors demonstrate that training variational networks on the synthesized data yields results comparable to training on raw k-space data. In a different study, Levac et al. [50] addressed the challenge of training MRI reconstruction models on heterogeneous data across multiple clients (data sites) while keeping the storage of individual scans local. The authors investigate an adaptive federated learning approach, where a global model is first trained across multiple clients without sharing any raw data between them, and then each client uses a small number of available datasets to fine-tune the global model. Numerical experiment results demonstrate that this approach can boost the performance of both under-represented clients, which participated in the federated training, and clients that were absent from it.

MRI scans can also be accelerated by reducing the number of scan repetitions, which are commonly required for improving the signal-to-noise ratio (SNR). Mohammadi et al. [51] propose a DL-based method for denoising low-SNR rectal cancer diffusion-weighted images (DWI) obtained with a high b-value. According to their method, DWI images acquired using a low b-value (characterized by high SNR) are used for guidance. The results, ranked using blind radiologist tests, indicate that the method enables an eight-fold scan time acceleration.

## 2. Image Synthesis and Parameter Quantification

The ability to derive meaningful tissue-characterizing images from raw data is yet another appealing application of AI in MRI. In this issue, Wu et al. designed a convolutional neural network (CNN) for the synthesis of water/fat images from dual- (instead of multi-) echo images [52]. In addition to the high fidelity shown in the output images, the proposed method demonstrated a 10-fold acceleration in computation time and a generalization ability to unseen organs and metal-artifact-containing images. In a different study, Zou et al. [53] proposed a manifold-learning framework that enables the reconstruction of free-breathing cardiac MRI data and the synthesis of cardiac cine movies. This framework enables the generation of synthetic breath-held cine movies with data on demand, e.g., movies with different inversion contrasts. Additionally, it enables the estimation of T_1_ maps with specific respiratory phases.

The accurate *quantification* of biophysical parameters is a long-sought-after goal in MRI. It is motivated by the superior reproducibility and improved diagnostic ability offered by distilled biological information. Traditionally, the derivation of parameter tissue maps required repeated acquisition in close to steady-state conditions, which yielded very long acquisition times. However, recently proposed frameworks for AI-based acquisition and quantification have rendered the *rapid* extraction of these parameters a viable option. A few such examples include the mapping of T_1_ and T_2_ relaxation times [54,55,56,57,58], semisolid magnetization transfer (MT) and chemical exchange saturation transfer (CEST) proton volume fraction and exchange rate [59,60,61,62,63], and susceptibility [64].

In this issue, Amer et al. combined quantitative T_2_ and proton density parameter maps with a multi-step classification pipeline aimed at segmenting and differentiating the various leg tissues [65]. By exploiting both fully and weakly supervised architectures, they were able to distinguish between the muscle, subcutaneous adipose, and infiltrated adipose tissues. Next, they exploited the resulting tissue areas for deriving a disease severity biomarker in muscle dystrophies. In another transverse relaxation rate quantification study, Lu et al. [66] designed and trained a cascade of two CNNs for image denoising and R2* mapping, and utilized it for iron-loaded liver relaxometry.

## 3. Automated Segmentation in Data-Challenging Regimes

AI techniques have recently led to state-of-the-art results in the automated segmentation of structure and pathology. For example, a significant body of work has been dedicated to the segmentation of brain tumors [67,68,69] and abdominal tissues/organs of interest [70,71,72]. Nevertheless, the development of AI techniques requires large training datasets, which are often scarce due to the high cost of data labeling. Moreover, the “off-label” use of other datasets could lead to biased results [73]. To overcome these hurdles, two papers investigated the benefits of pre-training segmentation networks on different datasets for solving different tasks. Dhaene et al. [74] proposed a method for automated segmentation of cardiac MRI (CMR) data, focusing on an MRI sequence that yields *tagged* MRI (which is useful for myocardial strain measurement). At present, publicly available tagged CMR datasets with myocardial annotations are scarce. The authors introduce a CycleGAN network that can transform cine data to synthetic tagged CMR data, and investigate the use of the synthetic data for training two segmentation networks. They show that pre-training the networks with the synthetic tagged-MRI data leads to faster convergence and better performance compared with training the networks from scratch. Their strategy achieves state-of-the-art results while using only a small dataset of real tagged CMR images.

Dominic et al. [75] suggest pre-training segmentation models on “pretext tasks”, where images are perturbed and the model is trained to restore them. They investigate two such tasks: context prediction, where random image pixels are set to zero, and context restoration, where image patches are randomly swapped. Their results demonstrate that pre-training increases the robustness of the segmentation models in limited labeled data regimes.

Another research direction that draws significant attention is end-to-end design of reconstruction and segmentation techniques. Although these two tasks are often addressed separately, there could be much benefit in solving them in tandem. This special issue includes a paper that summarizes the K2S challenge, which focused on this end-to-end approach and was hosted at the 25th International Conference on Medical Image Computing and Computer-Assisted Intervention (MICCAI) (Singapore, 2022) [76]. The challenge participants were required to submit DL models that can generate segmentation maps directly from 8x undersampled raw MRI measurements. The challenge organizers created a unique dataset consisting of 300 knee MRI scans, accompanied by radiologist-approved tissue segmentation labels. A total of twelve teams submitted their work to this challenge, and four of them obtained top performance. All the top submissions produced high-quality segmentation maps of knee cartilage and bone, which were suitable for downstream biomarker analysis. Interestingly, the organizers found no correlation between the reconstruction and segmentation metrics.

## 4. MRI Scan Planning

Automated scan prescription is an emerging AI application, which holds new prospects for clinical workflow optimization. At present, MRI scans necessitate a time-demanding manual prescription, based on human expertise. Two papers in this special issue propose novel techniques for automating this process. Lei et al. developed an automatic system for field-of-view (FOV) prescription using an intra-stack attention neural network [77]. The suggested system outperforms standard CNN models while producing prescriptions that were not significantly different than those produced by a radiologist. The method was validated using a challenging set of pediatric pelvic and abdominal images, where a typically large variance in body shape is expected. A radiologist confirmed the quality of the output segmentation maps, rating 69 out of the 80 examined images as clinically acceptable. The inference time was less than 0.5 s, rendering this approach as a promising tool for accelerating the clinical imaging pipeline.

Eisenstat et al. addressed the task of automated fetal MRI planning [78]. Determining the fetus’s presentation is an important element in the sequence planning, as it affects the mode of delivery. The authors designed a CNN-based architecture, dubbed Fet-Net, for the automatic classification of a 2D slice image into one of four presentation categories. Trained on 143 3D MRI datasets, the method’s performance was better than those of alternative methods.

## 5. Conclusions

This special issue includes seventeen papers that showcase the recent developments in harnessing AI to improve MRI workflow. The reported techniques involve various “intervention points” along the imaging pipeline, including protocol planning, data acquisition, image reconstruction, quantitative parameter mapping, and automated segmentation.

Another medical imaging regime where AI has brought considerable benefits is automated diagnosis and prognosis. AI has been found useful, for example, for the diagnosis of breast and prostate cancer from MRI [79,80], the diagnosis of COVID-19 from medical images [81,82], and fault detection in health management [83]. Furthermore, AI-based methods have led to state-of-the-art results in lesion detection and classification [84,85,86,87,88].

Two dominant trends can be identified in the papers published in this issue. First, the emergence of methods for addressing the lack or scarcity of open-access training data, a known obstacle for algorithm development [73]. Here, this challenge was addressed using data-style transfer [74], manifold learning directly from undersampled dynamic MRI data [53], complex-valued data synthesis with GANs [49], pre-training on “pretext tasks” [75], and federated learning [50]. The second trend is the shift towards more comprehensive AI pipelines, which aim to address more than one component of the MRI workflow. It includes frameworks that jointly optimize the sampling pattern and reconstruction [45] and techniques that generate segmentations directly from undersampled raw MRI measurements, thereby conducting both reconstruction and segmentation [76].

In summary, this issue serves as a further compelling evidence for the continuous contribution and promise of AI-based strategies for the MRI field. We expect that the upcoming years will see a consistent rise in the practical use of AI in medical imaging, with further impact on emerging applications, such as low-field MRI [89,90] and real-time MRI for MR-guided interventions [91,92,93].
